# Discovery and Characterization of Novel RNA Viruses in Aquatic North American Wild Birds

**DOI:** 10.3390/v11090768

**Published:** 2019-08-21

**Authors:** Marta Canuti, Ashley N. K. Kroyer, Davor Ojkic, Hugh G. Whitney, Gregory J. Robertson, Andrew S. Lang

**Affiliations:** 1 Department of Biology, Memorial University of Newfoundland, 232 Elizabeth Ave., St. John’s, NL A1B 3X9, Canada; 2 Animal Health Laboratory, Laboratory Services Division, University of Guelph, 419 Gordon St., Guelph, ON N1H 6R8, Canada; 3 Wildlife Research Division, Environment and Climate Change Canada, 6 Bruce Street, Mount Pearl, NL A1N 4T3, Canada

**Keywords:** avian viruses, metapneumovirus, coronavirus, calicivirus, virus discovery, novel viruses, viral epidemiology

## Abstract

Wild birds are recognized viral reservoirs but our understanding about avian viral diversity is limited. We describe here three novel RNA viruses that we identified in oropharyngeal/cloacal swabs collected from wild birds. The complete genome of a novel gull metapneumovirus (GuMPV B29) was determined. Phylogenetic analyses indicated that this virus could represent a novel avian metapneumovirus (AMPV) sub-group, intermediate between AMPV-C and the subgroup of the other AMPVs. This virus was detected in an American herring (1/24, 4.2%) and great black-backed (4/26, 15.4%) gulls. A novel gull coronavirus (GuCoV B29) was detected in great black-backed (3/26, 11.5%) and American herring (2/24, 8.3%) gulls. Phylogenetic analyses of GuCoV B29 suggested that this virus could represent a novel species within the genus *Gammacoronavirus*, close to other recently identified potential novel avian coronaviral species. One GuMPV–GuCoV co-infection was detected. A novel duck calicivirus (DuCV-2 B6) was identified in mallards (2/5, 40%) and American black ducks (7/26, 26.9%). This virus, of which we identified two different types, was fully sequenced and was genetically closest to other caliciviruses identified in Anatidae, but more distant to other caliciviruses from birds in the genus *Anas*. These discoveries increase our knowledge about avian virus diversity and host distributions.

## 1. Introduction

Among vertebrates, the class Aves (birds) is one of the most ubiquitous lineages on Earth and, with over 10,000 living species, is also one of the most diverse [1]. Birds occupy various habitats, from natural to urban environments, and serve diverse ecological roles in various ecosystems [2]. They have a global distribution and, thanks to their ability to fly long distances, are capable of migrating across biological and geographical borders and over broad spatial scales [1,3]. Because of these characteristics, birds play an important role in the dispersal of microbes and, therefore, influence microbial dynamics, impacting the ecology and evolution of various viruses and bacteria [3,4,5]. 

Birds are reservoir hosts for many bacteria and viruses, including avian pathogens and zoonotic agents, such as avian influenza A virus (AIV), *Borrelia burgdorferi,* West Nile virus, and antimicrobial-resistant bacteria [6,7,8]. In particular, RNA viral diversity can be high in the avian reservoirs, as exemplified by AIV [7] and coronaviruses [9]. Furthermore, recent virus discovery studies have highlighted how our knowledge about viral diversity in birds is still limited, with many novel RNA viruses discovered in different avian hosts [10,11,12]. Considering the vast geographic territory birds occupy and the richness of avian species, many more yet unknown viruses likely exist in birds in different environments.

In a previous study, we reported the identification of viral fragments from potentially novel avian RNA viruses belonging to the viral families *Pneumoviridae*, *Coronaviridae*, and *Caliciviridae* [13], all known to include avian viruses among their members.

Metapneumoviruses have spherical virions comprising a host-derived envelope containing spiking viral glycoproteins, and a nucleocapsid formed by viral RNA and proteins separated from the envelope by an intermediate protein layer known as the matrix [14]. Within the genus *Metapneumovirus* (family *Pneumoviridae*), the species *Avian metapneumovirus* (AMPV) is a paraphyletic group encompassing viruses known to infect birds. This species officially includes four subgroups (A to D) with AMPV-C being more closely related to viruses within the species *Human metapneumovirus* (HMPV) than to other AMPVs [14]. A recent study also reported the identification of a potentially novel subgroup [15]. Avian metapneumoviruses are significant poultry pathogens as they cause an acute respiratory disease in turkeys, known as rhinotracheitis, and are one of the factors involved in swollen head syndrome in chickens [16]. AMPV has a worldwide distribution, except for Australia where it has never been documented [17], but the various subgroups are distributed differently. For example, subtype C has a global distribution, but only two subtypes have ever been found in North America, AMPV-C and the newly identified and yet unclassified subtype [15,18]. AMPV RNA has been identified in various wild birds, such as ducks, geese, gulls, starlings, and others [15,18,19,20,21,22].

Viruses within the *Coronaviridae* subfamily *Orthocoronavirinae* have typically spherical enveloped virions from which viral glycoproteins (spikes) are projected, which surrounds a helical nucleocapsid. All currently known avian coronaviruses are included within the two genera *Gammacoronavirus* and *Deltacoronavirus* of this subfamily [23,24]. Although both genera include a variety of viruses of wild birds, the most studied avian coronaviruses are those within the genus *Gammacoronavirus*, as they are a cause for great concern for the poultry industry worldwide. In fact, the infectious bronchitis virus (IBV), a gammacoronavirus within the subgenus *Igacovirus*, can cause a severe respiratory disease in domestic fowl, associated sometimes with nephritis and urogenital complications, with significant mortality [25]. Viruses belonging to the same species as IBV (*Avian coronavirus*) have been found worldwide in other avian hosts, such as turkey, where coronaviruses cause severe enteritis [26], ducks, gulls, and others [9,24,27,28,29,30,31]. Finally, recent studies have discovered that other igacoviral species exist in birds [32,33,34].

Compared to other avian viruses, our knowledge about avian caliciviruses is still limited as their discovery was very recent. Caliciviruses have non-enveloped virions whose capsid, formed by the two structural proteins VP1 and VP2, encloses a molecule of single-stranded, positive-sense RNA [35]. The first avian caliciviruses were identified in poultry, but so far, no clear links with disease have been established [36,37,38,39]. Currently, avian caliciviruses are classified within the two genera *Bavovirus* (species: *Bavaria virus*) and *Nacovirus* (species: *Nacovirus A*), two viral clades that are monophyletic and whose closest relatives are members of the genus *Sapovirus* [35,37]. However, recent studies have determined that the diversity of avian caliciviruses is higher than anticipated and several novel viral species, and possibly genera, have been identified in various avian hosts, including geese, ducks, shorebirds, and others [12,40,41,42].

In this study, we fully sequenced a novel duck calicivirus and a novel gull metapneumovirus and obtained extended sequence information of a potentially novel gull coronavirus we discovered previously in wild birds with the ViDiT-CACTUS method [13]. Our objective was to molecularly characterize the novel viruses and investigate their molecular epidemiology within the respective avian populations in which they were identified.

## 2. Materials and Methods

### 2.1. Sample Collection

This study involved 50 samples collected from two gull species (24 American herring gull, *Larus argentatus smithsonianus*, and 26 great black-backed gull, *L. marinus*) and 33 samples collected from ducks (26 American black duck, *Anas rubripes*, 5 mallard, *A. platyrhynchos*, and 2 American black duck/mallard hybrids). All samples were collected within the city of St John’s, Newfoundland and Labrador, Canada. St. John’s is an isolated urban center, at the eastern edge of North America. Agriculture is limited in the region, but a modern (high biosecurity) poultry operation operates in the area. The local wild avifauna is typical of northern and productive marine areas, and aquatic species that are comfortable with human interaction (ducks and gulls) are abundant in the city. Gull samples were collected in 2015 at a regional landfill and ducks were sampled in 2015 and 2014 at two different ponds. This study was carried out in accordance with the recommendations of the Canadian Council on Animal Care. The protocol was approved by the Memorial University Institutional Animal Care Committee (approved protocols 13-01-AL and 14-01-AL). Live birds were captured under federal authority (Canadian Wildlife Service Migratory Bird Banding Permit 10559). Samples, all AIV-negative, were paired individual oropharyngeal and cloacal swabs (polyester swabs, Starplex Scientific), submerged together in 3 mL viral transport medium (Starswab Multitrans System, Starplex Scientific). Samples were kept frozen until processing.

### 2.2. Molecular Methods

During a previous study, sequence fragments of the three viruses were identified with the ViDiT-CACTUS virus discovery method [13]. ViDiT is an ultra-low-cost random-PCR-based library preparation method for metagenomic investigations that includes: An initial sample pre-treatment to eliminate unwanted background, a retro-transcription reaction to generate cDNA from RNA, an amplification with tailed random octamers, two final amplification rounds for library completion and enrichment, and Ion Torrent sequencing. Viral sequences identified with CACTUS, a Python-based wrapper script that handles PHRAP-based read assembly and BLASTn- and DIAMOND-based sequence taxonomy assignment, were used in this study as templates for primer design. To extend viral sequence information or obtain the complete viral genome, these primers were used to connect identified viral fragments by specific PCRs or to perform genome walking with the ViDiWa method as previously described [5], followed by Sanger sequencing. ViDiWa is a ViDiT-based nested-PCR method that uses tailed random octamers in combination with virus-specific primers to amplify unknown genomic regions flanking known sequences. An attempt was made to obtain the 5’ and 3’ termini of the novel viruses with 5’ and 3’ RACE (5’RACE System, 3´ RACE System, Thermo Fisher Scientific). Finally, the 5’ termini of the gull metapneumovirus was obtained by a PCR involving a strain specific primer and an equimolar mixture of primers designed based on conserved terminal sequences of other AMPVs (Meta_End1: GAATGTATACGGTTTTTTTGCCGT; Meta_End2: GGATGAATACGGTTTTTTTGCCGT; Meta_End3: TGATGAATACGGTTTTTTTCTCGT).

Isolated nucleic acids from a previous study [5] were used as templates for reverse transcription using SuperScript IV (Thermo Fisher Scientific, Waltham, MA, USA). Molecular screening was performed by hemi-nested PCRs with the primers listed in Table 1. The screening involved the amplification of 182 nt of the L gene of the gull metapneumovirus, 188 nt of the ORF 1a of the gull coronavirus, and 417 nt of the VP1 gene of the duck calicivirus. All PCR-positive samples were confirmed by sequencing. Additionally, an almost complete genome was obtained for another caliciviral strain, the entire VP2 ORF was sequenced for an additional 3 caliciviruses, one entire and three partial sequences of the F gene were sequenced for an additional 4 metapneumoviruses, and a 396–537 nt fragment of the viral polymerase gene was sequenced for 3 coronaviruses. All primers used in this study are available upon request.

### 2.3. Sequence and Phylogenetic Analyses

**Figure 1:**
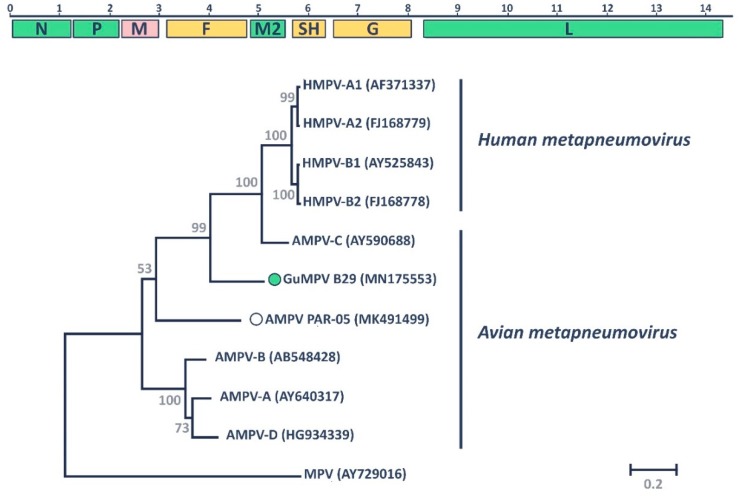
Molecular characteristics of GuMPV B29. The genome organization of the novel virus is depicted at the top. Identified open reading frames (ORFs) are illustrated by colored rectangles: Nonstructural proteins are in green (N: nucleoprotein; P: phosphoprotein, L: polymerase), envelope glycoproteins are in yellow (F: fusion protein; SH: small hydrophobic protein; G: glycoprotein), and the matrix protein (M) is in pink. The scale bar on top represents genomic position in Kb. The phylogenetic placement of GuMPV B29 within the genus *Metapneumovirus* based on the complete amino acid sequence of the L protein is shown at the bottom. The two species *Human metapneumovirus* (AMPV) and *Avian metapneumovirus* (AMPV) are indicated on the right, and the murine pneumovirus (MPV) was used as an outgroup. Accession numbers are indicated in parentheses. Officially assigned viral sub-groups are indicated by letters (A1-2, B1-2 for HMPV, A–D for AMPV), while recently identified viruses that lack official taxonomic designations are indicated by circles with the virus identified in this study indicated by the filled green circle. The tree was built with the maximum likelihood method [47] using MEGA 7 [43] based on the Le Gascuel model [49], identified as the best-fitting model by the model test in MEGA. A discrete Gamma distribution was used to model evolutionary rate differences among sites, branch lengths are proportional to genetic distances as indicated by the scale bar, and the outcome of the bootstrap analysis [48] is shown next to the nodes.

**Figure 2:**
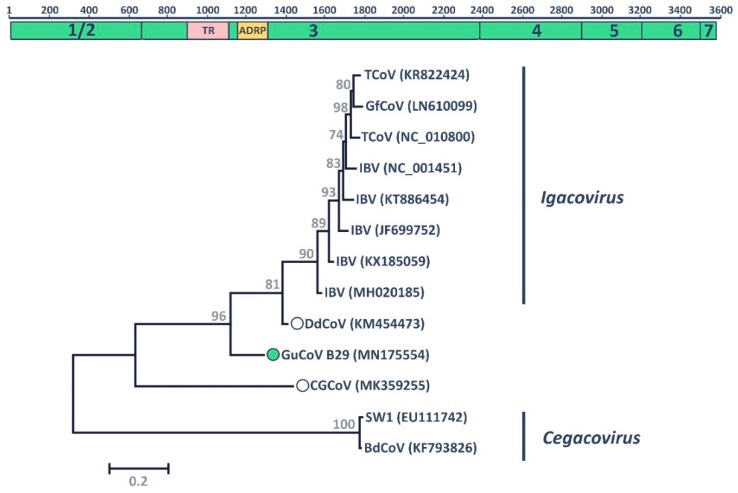
Molecular characteristics of GuCoV B29. The organization of the partial 1a polyprotein of the novel virus is depicted at the top. Identified mature peptides (non-structural proteins, nsp1/2–7) are illustrated by green rectangles. The pink rectangle indicates the area in nsp3 where the tandem repeat (TR) was identified, while the yellow rectangle shows the location of the conserved ADP-ribose 1-“phosphatase (ADRP) replicase domain within nsp3. The scale bar on top represents the amino acid position within the polyprotein. The phylogenetic placement of GuCoV B29 within the genus *Gammacoronavirus* based on the concatenated alignments of the ADRP and 3C-like proteinase (3CL^pro^) (corresponding to nsp5) is shown at the bottom. The two species *Igacovirus* and *Cegacovirus* are indicated on the right. Accession numbers of sequences used (SW1: Beluga whale coronavirus; BdCoV: Bottlenose dolphin coronavirus; IBV: Infectious bronchitis virus; TCoV: Turkey coronavirus; GfCoV: Guinea fowl coronavirus; DdCoV: Dominant-duck coronavirus; GuCoV: Gull coronavirus; CGCoV: Canada goose coronavirus) are indicated in parentheses. Recently identified viruses that lack official taxonomic designations are indicated by circles with the virus identified in this study indicated by the filled green circle. The tree was built with the maximum likelihood method [47] using MEGA 7 [43] based on the Le Gascuel model [50], identified as the best-fitting model by the model test in MEGA. A discrete Gamma distribution was used to model evolutionary rate differences among sites, branch lengths are proportional to genetic distances as indicated by the scale bar, and the outcome of the bootstrap analysis [48] is shown next to the nodes.

**Figure 3:**
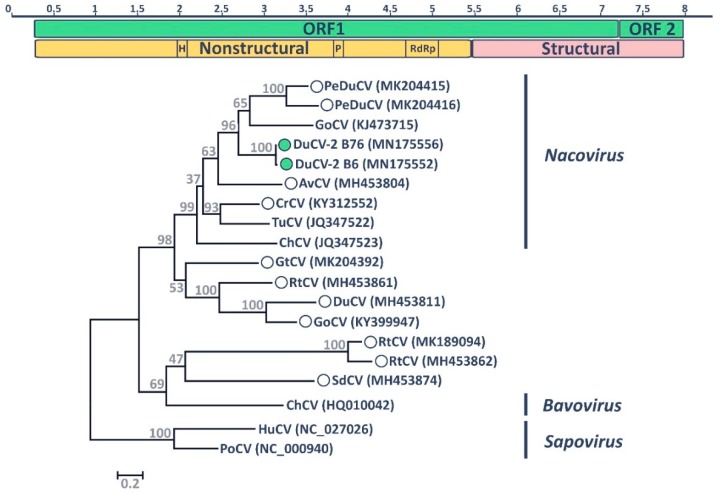
Molecular characteristics of DuCV-2 B6. The genome organization of the novel virus is depicted at the top. The two identified ORFs are illustrated by green rectangles, while nonstructural and structural protein regions are indicated in yellow and pink, respectively. Enzymatic domains typical of caliciviruses are indicated (H: helicase/NTPase; P: 3C-like cysteine protease; RdRp: RNA-dependent RNA polymerase) and the scale bar above indicates genomic position in Kb. The phylogenetic placement of DuCVs-2 B6 and B76 within the genus *Nacovirus* based on the predicted amino acid sequence of the major capsid protein (VP1) is shown at the bottom. Hosts of viruses used for phylogenetic reconstruction are indicated within the strain designation (PeDu: Pink-eared duck; Du: Duck; Go: Goose; Av: Avocet; Cr: Crane; Tu: Turkey; Ch: Chicken; Gt: Grey teal; Rt: Ruddy turnstone; Sd: Shelduck; Hu: Human; Po: Porcine), while accession numbers of sequences are indicated in parentheses. Viral genera are depicted on the right and the two mammalian caliciviruses of the genus *Sapovirus* were used as an outgroup. Recently identified viruses that lack official taxonomic designation are indicated by circles and the viruses identified in this study are indicated by filled green circles. The tree was built with the maximum likelihood method [47] using MEGA 7 [43] based on the Le Gascuel model [50], identified as the best-fitting model by the model test in MEGA. A discrete Gamma distribution was used to model evolutionary rate differences among sites, branch lengths are proportional to genetic distances as indicated by the scale bar, and the outcome of the bootstrap analysis [48] is shown next to the nodes.

Geneious R11 (Biomatters) was used for sequence visualization, assembly, genomic annotations, and ORF and protein sequence predictions. For comparisons and phylogenetic analyses, three different datasets were built for each novel virus. For metapneumoviruses, representative members of each subgroup within the species *Avian metapneumovirus* and *Human metapneumovirus* [14,15] were used and the *Murine orthopenumovirus* was used as an outgroup. For coronaviruses, representative members of the species *Igacovirus* identified in various avian hosts as well as viruses recently proposed as novel species were included in the analyses [23,32,33] and the two viruses infecting marine mammals within the species *Cegacovirus* were used as an outgroup. For caliciviruses, all avian viruses identified to date were included in the tree [35,41,42], excluding only strains that were >99% identical to other viruses already included, and two sapoviruses of mammals (human calicivirus and porcine calicivirus) were included as an outgroup. Accession numbers of sequences used are provided in Figure 1, Figure 2 and Figure 3.

Phylogenetic analyses were performed with MEGA 7 [43] on protein sequence alignments generated with MAFFT version 7 [44], as implemented in Geneious, and processed with Trim Al 1.3 [45], implemented on the Phylemon 2.0 webserver [46], to polish poorly aligned regions. The maximum likelihood method [47] was used for phylogenetic inference and the best model for sequence distance estimation was determined with a model test in MEGA 7. Finally, 1000 bootstrap replicates were used to evaluate node robustness [48]. Mean percentage identities (1–p-distance) between groups were calculated with MEGA 7.

### 2.4. Data Availability 

All sequences obtained in this study have been deposited in GenBank under accession numbers MN175552–MN175554 for complete genomes and MN175555–MN175568 for partial sequences.

## 3. Results and Discussion 

During a previous study, we identified genomic fragments belonging to potentially novel RNA viruses [13]. Using a genome-walking technique, we obtained the complete coding sequence of a novel metapneumovirus (gull metapneumovirus or GuMPV, strain B29) and a novel calicivirus (duck calicivirus 2 or DuCV-2, strain B6), and obtained approximately 10 Kb of a genomic sequence of a potentially novel coronavirus (gull coronavirus or GuCoV, strain B29). These are each discussed in more detail below.

### 3.1. Gull Metapneumovirus 

The novel GuMPV B29 was identified, in a co-infection with the novel GuCoV B29, in a great black-backed gull sampled in the city of St John’s in 2015. Unfortunately, we only managed to obtain the complete coding sequence of this virus. The 5’ RACE experiments were unsuccessful and 3’ RACE was not possible because metapneumoviruses possess a single-stranded, negative-sense RNA genome [14] that lacks 3’ polyadenylation. However, taking advantage of the high conservation of the 5’ trailer sequence of AMPVs, we managed to determine most of the 5’ untranslated region (UTR), but the 3’ UTR remains mostly unknown.

The obtained sequence was approximately 14.5 Kb, larger than the genomes of both HMPVs (13.2–13.4 Kb) and other AMPVs (13.5–14 Kb), but similar in size to the genome of AMPV subtype C. As shown in Figure 1, consistent with other MPVs, the genome of GuMPV B29 contains nine open reading frames (ORFs), coding for the three nucleocapsid-associated proteins (nucleoprotein: N; phosphoprotein: P; large polymerase: L), three glycosylated transmembrane surface envelope proteins (fusion protein: F; attachment protein: G; small hydrophobic protein: SH), two non-glycosylated membrane or matrix proteins (M and M2-1), and the small M2-2 protein. With the exception of the ORFs of M2-1 and M2-2 that partially overlap (indicated together as M2 in Figure 1), all other genes were separated by short intergenic regions with variable length (25–175 nt) [14].

When compared to other metapneumoviruses, most of the predicted proteins of GuMPV B29 were similar in size to those of other human and avian viruses, except for the SH protein that possessed an additional C-terminal 40 amino acids. Protein sizes were conserved among all metapneumoviruses, except for the G protein, which is significantly larger in avian compared to human viruses. Consistent with what was previously reported [14], most proteins showed a high degree of conservation among all MPVs, except for the highly variable G and SH proteins (Supplementary Figure S1). As for other AMPVs, the typical trypsin-dependent cleavage motif (RQSR) within the F protein, which allows the inactive F0 protein to be proteolytically activated into the fusion competent F1 and F2, was lacking, suggesting alternative cleavage mechanisms [50]. Finally, the four core metapneumoviral motifs essential for polymerase activity were well conserved among all avian viruses (motif A: SIVTDLSKFNQAF; motif B: GLYR[F/Y]HMGGIEGWCQK[M/L]WTMEAISLL; motif C: SLLNGDNQSI; motif D: QSEGVM[Y/H]PXXIK[K/R][V/I]) [51].

Phylogenetic analysis of the conserved L proteins of representative members of all currently known AMPV subgroups, plus the *Murine orthopneumovirus* that was used as an outgroup, showed that the novel GuMPV B29 falls within the species *Avian metapneumovirus* (Figure 1). Like the other recently described novel AMPV (AMPV PAR-05), GuMPV B29 was located between the clade that includes HMPVs and AMPV-C and the clade including AMPVs-A, -B, and -D. However, while AMPV PAR-05 was more closely related to the other avian viruses (average pairwise identity: 66.6%), GuMPV B29 seemed to be more closely related to the other clade (average pairwise identity: 69.3% vs. 65%) (Supplementary Figure S1). Similar results were obtained with the other genomic regions (Supplementary Figure S1). Therefore, while the complete genomic characterization of the novel GuMPV B29 virus supports its classification within the species *Avian metapneumovirus*, protein sequence comparisons and phylogenetic analyses demonstrate that it is clearly distant from all other known subgroups and it can be considered the first member of a new subgroup. Our discovery therefore increases the number of known AMPV subgroups to six.

We evaluated the distribution of GuMPV B29 within the local gull population by screening a total of 50 samples collected in September and October 2015 at the same location. Overall, 10% (*N* = 5) of the samples were positive: Four positive samples were collected from great black-backed gulls (4/26, 15.4%) and one sample (1/24, 4.2%) from an American herring gull. One GuMPV–GuCoV co-infection was found. The complete sequence of the F gene was obtained for the virus infecting the herring gull and this was 99.8% identical to the strain identified in the original great black-backed gull. Three mutations, two of which were synonymous, distinguished the two strains. However, the same mutations were found in another partial F sequence obtained for a virus recovered in another great black-backed gull, while the remaining two partial F sequences were 100% identical to the original strain. Thus, we concluded that all of these gulls were infected with the same viral type.

To date, only a few studies have investigated the presence of AMPVs in gulls. While Turpin et al. could not find any serological evidence for AMPV infection in three species (American herring gull, ring-billed gull (*L. atricilla*), and laughing gull (*L. delawarensis*)) in the south-eastern United States [52], this was found in Germany in the European herring gull (*L. argentatus argentatus*) [53]. Furthermore, AMPV RNA was identified in a few ring-billed gulls found dead on a Minnesota turkey farm with a known history of AMPV [19], while HMPV-C was found in 22 European common gulls (*L. canus*) [20]. Our study further confirms gulls as AMPV hosts and indicates great black-backed and American herring gulls are additional host species. We also demonstrate that these viruses can spread among co-existing gull species and highlight how gulls may harbor a hidden viral diversity that deserves further investigation.

### 3.2. Gull Coronavirus

The novel GuCoV B29 was identified, in co-infection with the novel GuMPV B29, in a great black-backed gull sampled in the city of St John’s in 2015. Typically, the genome of avian coronaviruses is about 27–32 Kb in length and includes two short UTRs and six main ORFs, including those for the replicase gene on the 5’ end (the two overlapping 1a and 1b ORFs), and those for the structural proteins on the 3’ end, plus several accessory genes [23]. Unfortunately, we were only able to obtain a partial genomic sequence (approximately 10 Kb), which encompassed almost the entire sequence of the 1a ORF. Further attempts to extend the sequence, including genome walking and 5’ RACE experiments, were unsuccessful. The replicase gene is normally translated into a polyprotein that is post-transcriptionally processed by virus-encoded proteases into 15 mature non-structural proteins (nsps) [23]. The partial sequence obtained for GuCoV B29 encompassed the first six proteins (nsp1/2 through nsp7) and, by exploiting the relatively high conservation of the polyprotein sequence, we were able to identify putative cleavage sites [34,54,55,56] and predict the sequence of the mature peptides (Figure 2). Finally, within the obtained sequences, we were able to localize two of the seven conserved replicase domains used for viral classification: The ADP-ribose 1-“phosphatase (ADRP) domain in nsp3 and the 3C-like proteinase (3CL^pro^), which corresponds to nsp5 [57,58,59].

Interestingly, similar to what was previously identified in the duck-dominant coronavirus (DdCoV) [34], we identified a tandem repeat within the nsp3 ORF (indicated as TR in Figure 2). This region was 621 nt long and included nine copies, 94.2–100% identical to each other, of a 69 nt sequence (Supplementary Figure S2). The resulting 23 aa sequence repetition was predominantly composed of glutamic acid, lysine, proline, and glutamine, and was 69.6% identical to the one found in the DdCoV.

Members of the family *Coronaviridae* are classified based on the phylogeny of seven well-conserved replicase domains concatenated in one alignment [23]. Because only a partial genomic sequence was obtained, we could evaluate the relationship between GuCoV B29 and other members of the genus Gammacoronavirus using only the ADRP and 3CL^pro^ domains. The obtained phylogenetic tree (Figure 2) included the two main clades representing the two currently defined gammacoronaviral subgenera, one of the viruses infecting marine mammals (*Cegacovirus*) and the other viruses infecting birds (*Igacovirus*). GuCoV B29 was located between these two clades, similar to two other recently discovered avian coronaviruses, the DdCoV and the Canada goose coronavirus (CGCoV), both proposed as novel species within the genus [33,34]. This tree topology was conserved when phylogenetic analyses were performed with the whole available polyprotein sequence, but not when the two replicase domains were used separately (Supplementary Figure S3). In fact, within the ADRP domain, GuCoV B29 and DdCoV formed a highly supported clade. When the 3CL^pro^ region was used, DdCoV was included within the main *Igacovirus* clade, while GuCoV B29 still clustered separately (Supplementary Figure S3). This was probably due to the recombinant nature of DdCoV [34].

The closest relative to GuCoV B29 was the DdCoV, with 74% amino acid identity over the whole available sequence and 76.9% identity when considering the two concatenated replicase domains. Although the complete genomic sequence must be obtained to define a novel species, it is possible that GuCoV B29 represents the first member of a novel gammacoronaviral species within the genus *Igacovirus*, as viruses that share less than 90% amino acid identity in the conserved replicase domains are considered separate species and viruses that share more than 45% amino acid identity in those domains are considered members of the same genus.

We studied the distribution of GuCoV B29 within the same gull population investigated for GuMPV B29. Of the 50 screened gulls, five (10%) resulted positive: Three positive samples were collected from great black-backed gulls (3/26, 11.5%) and two samples (2/24, 8.3%) from American herring gulls. One AMPV–GuCoV co-infection was found. For two viruses identified in black-back gulls and one from herring gulls, we obtained 537 nt and 396 nt sequences within the nsp3 region, respectively. These sequences were 99.5–100% identical to the B29 strain and only one synonymous substitution was found, indicating that the same virus circulates in both gull species, as previously reported for other viruses in the same populations [5].

Gammacoronaviruses have already been identified with a positivity rate of approximately 1–8% in various gull species (black-headed gull (*L. ridibundus*), glaucous-winded gull (*L. glaucoscens*), vega gull (*L. argentatus vegae*), and glaucous gull (*L. hyperboreus*)) in the Bering Strait area [31], while other gamma- and deltacoronaviruses have been reported in several other studies in black-headed gulls in Scandinavia and Poland [60,61,62] and in a few other gull species (European herring gull and lesser black-backed gull (*L. fuscus*)) in Finland [61]. However, other studies did not detect any coronaviruses in American and European herring, lesser and great black-backed, black-headed, laughing, common/mew, and black-tailed (*L. crassirostris*) gulls in the United States [63], England [30], Scandinavia [61,62], Poland [60], and Korea [64]. To our knowledge, this is the first study that identified coronaviruses in great black-backed and American herring gulls, maybe indicating that different species of coronaviruses circulate in these birds. Furthermore, we demonstrate that the novel GuCoV B29, although circulating at low frequencies, can be transmitted between different co-existing gull species.

### 3.3. Duck Calicivirus

The novel calicivirus DuCV-2 B6 was found, in co-infection with a novel parvovirus [65], in a mallard sampled in the city of St John’s in 2015. The genomes of avian caliciviruses are 7.4–8.3 Kb in length and contain two ORFs, with the non-structural proteins encoded in the 5′ terminus of the genome and the structural proteins in the 3′ terminus [35]. For the DuCV-2 B6, we were able to obtain the complete coding sequence and, using 3’ RACE, the full 3’ terminal sequence (Figure 3). However, 5’ RACE was unsuccessful, and the 5’-terminal sequence remains undetermined. The obtained sequence was approximately 8.2 Kb and DuCV-2 B6 presented a genomic structure similar to what has been reported for other avian caliciviruses, with two main ORFs, the first one encoding a large polyprotein that undergoes cleavage by a virus-encoded protease to generate several mature nonstructural proteins as well as the major structural protein VP1, and the second one coding for the structural protein VP2 [35]. Additionally, we obtained a partial genomic sequence, covering 90% of the polyprotein sequence and the full VP2 sequence, of an additional strain detected in an American black duck (DuCV-2 B76).

Although polyproteins were highly variable across avian caliciviruses and it was not possible to determine with certainty the cleavage sites between the nonstructural proteins, we were able to identify the typical amino acid motifs associated with enzymatic functions in caliciviral nonstructural proteins [38,66,67]. The helicase/NTPase motif GXPGXGKT was identified in all avian caliciviruses, except for one of the turnstone caliciviruses (MH453861), and was identical in both of our sequenced strains (GPPGIGKT). The 3C-like cysteine protease motif G(D/Y)CGXP was highly conserved in all avian viruses and identical in the two sequenced strains (GDCGLP). Finally, we were able to identify the three RNA-dependent RNA polymerase (RdRp) motifs: DYSKWDST, which was present in most viruses as DY(S/K)(K/G)WDST and in the two DuCVs-2 as DYKKWDST, and GLPSG and YGDD, which were 100% conserved among all viruses. The position of these domains (Figure 3) suggested that the order of the proteins in the polyprotein is the same as for other caliciviruses [35].

A putative cleavage site for the VP1 capsid protein (E^1727^/G^1728^) was identified by comparing our obtained sequences with previously predicted cleavage sites [68] and confirmed by checking site conservation across strains. The putative VP1 protein (584 aa in size) originating from this cleavage site was used for phylogenetic inference. The novel identified viruses (DuCV-2 B6 and B76) clustered within one of the caliciviral clades that includes avian viruses, specifically in a clade that also includes the goose, chicken, and turkey caliciviruses classified within the genus *Nacovirus* (Figure 3). Specifically, the DuCV-2 sequences are included in a highly supported clade that includes the nacovirus goose calicivirus and two viruses recently reported in pink-eared ducks (*Malacorhynchus membranaceus*) [42] (within clade pairwise identity 45–97%). Overall, two main groups of avian viruses could be observed in the tree and the biggest of these clades, including 13 viruses, among which were those classified as nacoviruses, comprised viruses that were more closely related to each other than to other viruses (average within-group pairwise identity 46.9% vs. average between-groups identity 31.8%), suggesting they could represent a unique genus [35]. Interestingly, viruses identified in the bird family Anatidae (ducks and geese) could be observed in various branches of the tree (pink eared duck: PeDuCV; duck: DuCVs; goose: GoCVs; grey teal: GtCV; shelduck: SdCV). Phylogenetic trees built with the other genomic regions (VP2 and the nonstructural region of the viral polyprotein, Supplementary Figure S4) showed the same clade of anatine viruses composed of DuCVs-2, GoCV, and PeCVs, although bootstrap support for this clade was low in the VP2 tree.

We evaluated the distribution of DuCV-2 in two different duck populations from two different ponds in St John’s. In total, we screened 33 samples and nine of those (27.3%) were positive for DuCV-2; three of those were in co-infection with other DNA viruses (two with duck papillomaviruses [5] and one with a novel parvovirus [65]). Viruses were present at both sampled locations with positive rates ranging between 21.4% (3/14) and 31.6% (6/19) and viruses were found both in mallards (2/5, 40%) and American black ducks (6/26, 23.1%), as well as in a mallard/American black duck hybrid (1/2, 50%). For one of the viruses identified in a black duck, we obtained an almost complete coding sequence and, overall, the two DuCV-2 sequences shared 94.6% nucleotide identity, with ORFs 1 and 2 being 94.4% and 95.6% identical, respectively. For three additional viruses, all from black ducks, we obtained the full VP2 ORF sequence and sequences from black ducks were 99.3–99.8% identical to each other and 95.6–95.8% identical to the sequence from mallards.

Unfortunately, presumably due to low viral load, we could not obtain sequence data beyond the screening amplicon (360 nt) for the other viruses. In total, we observed two different circulating viral types, one type was found in all mallards, one black duck, and one of their hybrids and in both sampled locations, while the other was found exclusively in black ducks and only at one of the two sampled ponds. No differences were observed between the two different sampled years. The two variants differed by nine amino acids within VP2, six within the nonstructural part of the polyprotein and 19 within VP1. However, 10 of these variable sites in VP1 were due to polymorphisms within the black duck virus itself, highlighted by the presence of double-peaks in the electropherograms, indicating either intra-host mutations or co-infection by two or more strains. Although we have only limited information and more extended screening is necessary, these results indicate that similar viruses are circulating in co-existing duck populations, as previously reported for other viruses in the same duck populations [5].

To our knowledge, caliciviruses have been identified only twice in ducks (genus *Anas*) before. One virus was identified in a pool of samples from different individuals and another one was identified in a grey teal (*A. gracilis*) and both samples were collected from birds in Southern Australia [12,42]. These two viruses, according to our analyses, are not the closest relative to DuCV-2 B6, suggesting the presence of several different viral lineages in ducks. Furthermore, avian caliciviruses have been identified in China [40,68,69], Germany [38,39], Australia [12,42], and Brazil [41] and, to the best of our knowledge, this is the first report of avian caliciviruses in wild birds of North America.

## 4. Conclusions

We describe here the characterization and molecular epidemiology of three novel viruses belonging to three different viral families that were identified in four species of wild birds. Although follow-up studies are required to determine additional aspects of their ecology and distribution, the discovery of these viruses, divergent from currently known avian strains and representing novel taxa, increases our knowledge about avian virus diversity and host ranges. Each one of the viruses identified in this study was observed in different related bird species, indicating limited host-specificity. Our results highlight one more time the limited knowledge about avian and, more generally, animal virology and show how a multitude of yet unknown viruses exist, even in species that are frequently subjects of metagenomic investigations.

## Figures and Tables

**Table 1:** Primers used for molecular screening.

Primer	Sequence (5’–3’)	nt Position	Reference Genome
Gull metapneumovirus			
Meta_F15	TCAGATGGGTCTTCAAAGGTG	11957–11977	MN175553
Meta_F16	GCATAGACTGTCTGTCAGTAG	12084–12104	
Meta_R20	TACTCGTTGCACTGACTCCG	12246–12265	
Gull coronavirus			
Corona_F4	CTGTTGAGGTTGACGAACAAGG	1828–1849	MN175554
Corona_R5	AGTAACAGTCTTACCACCAGC	2040–2060	
Corona_ R4	GCACCAACTTGCGACATTGG	2001–2020	
Duck calicivirus			
Calici_F8	GATCTGGCATGTATGGAGGC	5851–5870	MN175552
Calici_F7	TCCTTCCACCAGGCATCAAC	5887–5906	
Calici_R13	GGTAGTGGTTCCAGGAGTAG	6284–6303	

## Supplementary Materials

The following are available online at https://www.mdpi.com/1999-4915/11/9/768/s1, Figure S1: Phylogenetic analyses of metapneumoviruses, Figure S2: Alignment of the 207 amino acid tandem repeat identified in the nsp3 region of the 1a ORF of GuCoV B29. Figure S3: Phylogenetic analyses of gammacoronaviruses, Figure S4: Phylogenetic analyses of avian caliciviruses.
