# Supplementary material for: Discovery and Characterization of Novel RNA Viruses in Aquatic North American Wild Birds
Source: Viruses. 2019 Aug 21;11(9):768. doi: 10.3390/v11090768 (PMC6784231; doi:10.3390/v11090768)
Supplement: Supplementary file 1 [file viruses-11-00768-s001.pdf]

Discovery and characterization of novel RNA viruses in aquatic North American wild birds

Marta Canuti, Ashley N. K. Kroyer, Davor Ojkic, Hugh G. Whitney, Gregory J. Robertson, Andrew S. Lang

Table of content

Supplementary Figure S1. Phylogenetic analyses of metapneumoviruses. 2

Supplementary Figure S2. Alignment of the 207 amino acid tandem repeat identified in the nsp3 region of the 1a ORF of GuCoV B29...3

Supplementary Figure S3. Phylogenetic analyses of gammacoronaviruses. 5

Supplementary Figure S4. Phylogenetic analyses of avian caliciviruses. 7

References...9

Supplementary Figure S1. Phylogenetic analyses of metapneumoviruses. The trees are based on full amino acid sequences of each viral protein of representative members of viruses within the genus *Metapneumovirus* (HMPV: species *Human metapneumovirus*; AMPV: species *Avian metapneumovirus*). For each tree the protein used for phylogenetic reconstruction is indicated on the top-left (N: nucleoprotein; P: phosphoprotein; M: matrix protein; F: fusion protein; SH: small hydrophobic protein; G: glycoprotein; L: polymerase). Officially assigned viral sub-groups are indicated by letters (A1-2, B1-2 for HMPV, A-D for AMPV), while recently identified viruses that lack official taxonomic designation are indicated by circles with the virus identified in this study (GuMPV B29) indicated by the filled green circle. In each tree the amino acid identities of GuMPV B29 compared to the two different viral groups included in color-coded boxes are indicated. The trees were built with the maximum likelihood method (Felsenstein, 1981) using MEGA 7 (Kumar et al., 2016) based on the best-fitting model identified by the model test in MEGA (F, L, M, M2-2: LG model (Le and Gascuel, 2008); G, M2-1, N, SH: JTT model (Jones et al., 1992); P: WG model (Whelan and Goldman, 2001)). A discrete Gamma distribution was used to model evolutionary rate differences among sites (for all trees except for M2-2) and the rate variation model allowed for some sites to be evolutionarily invariable (for G, L, M2-2). Branch lengths are proportional to genetic distances as indicated by the scale bar, and the outcome of the bootstrap analysis (Felsenstein, 1985) is shown next to the nodes.

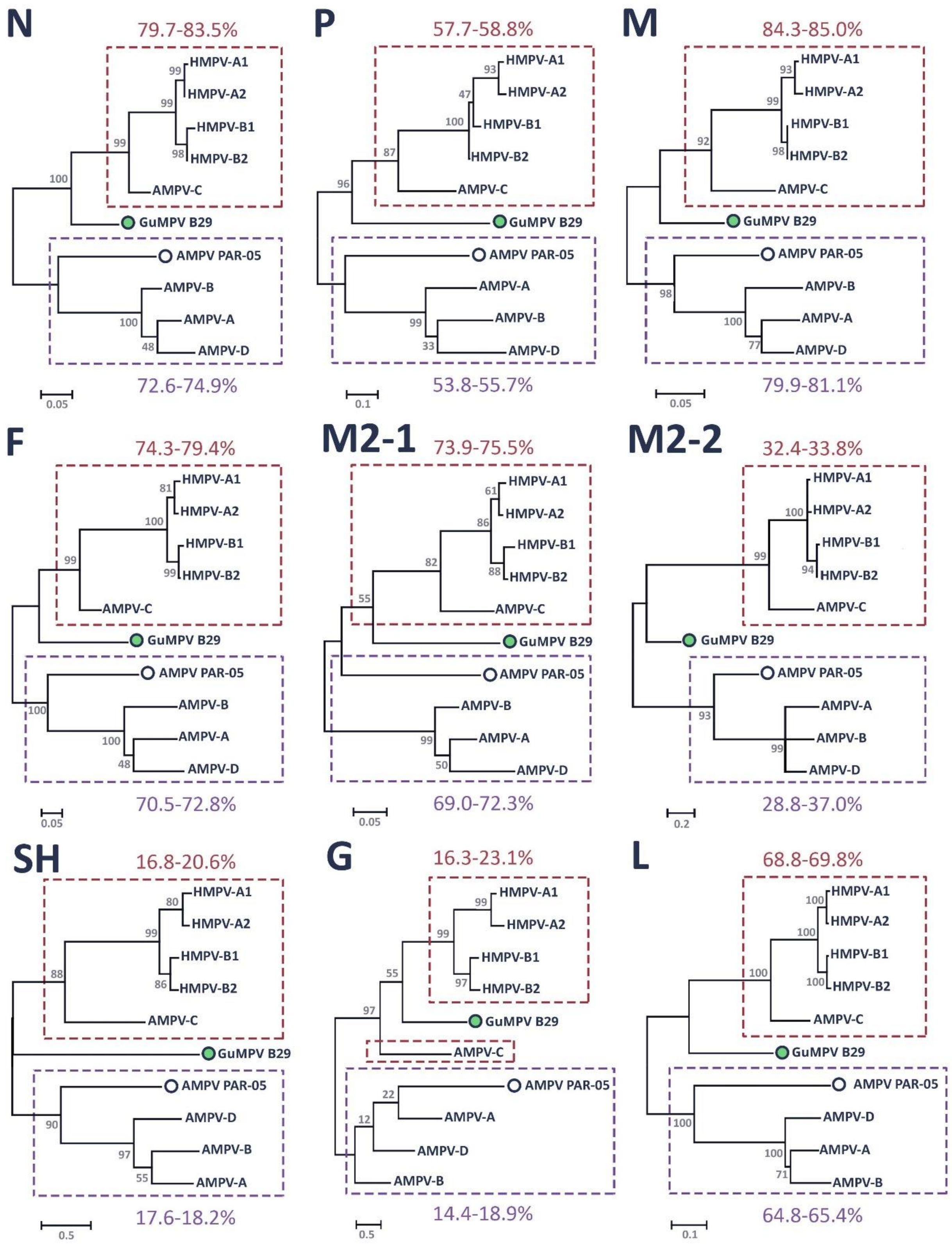
N
79.7-83.5%
HMPV-A1
HMPV-A2
HMPV-B1
HMPV-B2
AMPV-C
GuMPV B29
AMPV PAR-05
AMPV-B
AMPV-A
AMPV-D
99
99
99
98
100
100
48
0.05
72.6-74.9%
P
57.7-58.8%
HMPV-A1
HMPV-A2
HMPV-B1
HMPV-B2
AMPV-C
GuMPV B29
AMPV PAR-05
AMPV-A
AMPV-B
AMPV-D
93
47
100
87
96
99
33
0.1
53.8-55.7%
M
84.3-85.0%
HMPV-A1
HMPV-A2
HMPV-B1
HMPV-B2
AMPV-C
GuMPV B29
AMPV PAR-05
AMPV-B
AMPV-A
AMPV-D
93
99
98
92
98
100
77
0.05
79.9-81.1%
F
74.3-79.4%
HMPV-A1
HMPV-A2
HMPV-B1
HMPV-B2
AMPV-C
GuMPV B29
AMPV PAR-05
AMPV-B
AMPV-A
AMPV-D
81
100
99
99
100
100
48
0.05
70.5-72.8%
M2-1
73.9-75.5%
HMPV-A1
HMPV-A2
HMPV-B1
HMPV-B2
AMPV-C
GuMPV B29
AMPV PAR-05
AMPV-B
AMPV-A
AMPV-D
61
86
88
82
55
99
50
0.05
69.0-72.3%
M2-2
32.4-33.8%
HMPV-A1
HMPV-A2
HMPV-B1
HMPV-B2
AMPV-C
GuMPV B29
AMPV PAR-05
AMPV-A
AMPV-B
AMPV-D
100
99
94
93
99
0.2
28.8-37.0%
SH
16.8-20.6%
HMPV-A1
HMPV-A2
HMPV-B1
HMPV-B2
AMPV-C
GuMPV B29
AMPV PAR-05
AMPV-D
AMPV-B
AMPV-A
80
99
86
88
90
97
55
0.5
17.6-18.2%
G
16.3-23.1%
HMPV-A1
HMPV-A2
HMPV-B1
HMPV-B2
GuMPV B29
AMPV-C
AMPV PAR-05
AMPV-A
AMPV-D
AMPV-B
99
99
97
55
97
22
12
0.5
14.4-18.9%
L
68.8-69.8%
HMPV-A1
HMPV-A2
HMPV-B1
HMPV-B2
AMPV-C
GuMPV B29
AMPV PAR-05
AMPV-D
AMPV-A
AMPV-B
100
100
100
100
100
100
71
0.1
64.8-65.4%

Supplementary Figure S2. Alignment of the 207 amino acid tandem repeat identified in the nsp3 region of the 1a ORF of GuCoV B29. Each 23 amino acid long repetitive sequence is identified by a number (1 to 9) that corresponds to its position in the genome. Both the nucleotide and the corresponding predicted amino acid sequences are provided as consensus sequences; agreements with these sequences are indicated by dots, while variations identified in each repeat are indicated by one or three-letter codes for nucleotides and amino acids, respectively.

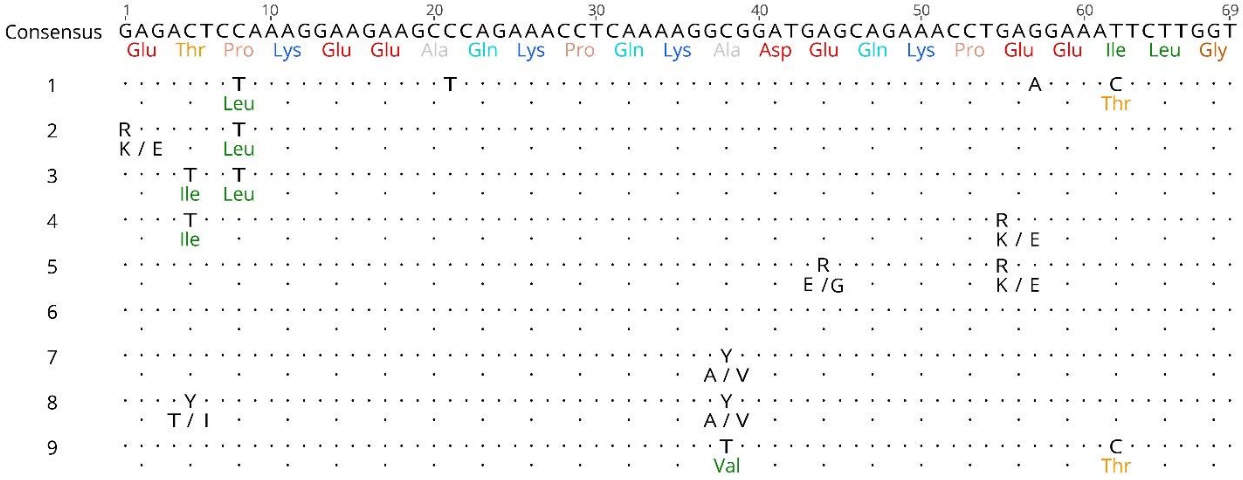

Supplementary Figure S3. Phylogenetic analyses of gammacoronaviruses. The trees are based on amino acid sequences of the two conserved replicase domains (ADRP and $3CL^{pro}$) and the whole available 1a ORF of viruses within the genus *Gammacoronavirus* (SW1: beluga whale coronavirus; BdCoV: bottlenose dolphin coronavirus; IBV: infectious bronchitis virus; TCoV: turkey coronavirus; GfCoV: Guinea fowl coronavirus; DdCoV: dominant-duck coronavirus; GuCoV: gull coronavirus; CGCoV: Canada goose coronavirus). Recently identified viruses that lack official taxonomic designation are indicated by circles with the virus identified in this study (GuCoV B29) indicated by a filled green circle. In each tree the amino acid identities of GuCoV B29 compared to the three different avian viral groups included in color-coded boxes are indicated. The trees were built with the maximum likelihood method (Felsenstein, 1981) using MEGA 7 (Kumar et al., 2016) based on the best-fitting model identified by the model test in MEGA ($3CL^{pro}$ and ADRP: LG model (Le and Gascuel, 2008); 1a: JTT model (Jones et al., 1992)). A discrete Gamma distribution was used to model evolutionary rate differences among sites and the rate variation model allowed for some sites to be evolutionarily invariable (for ADRP and 1a). Branch lengths are proportional to genetic distances as indicated by the scale bar, and the outcome of the bootstrap analysis (Felsenstein, 1985) is shown next to the nodes.

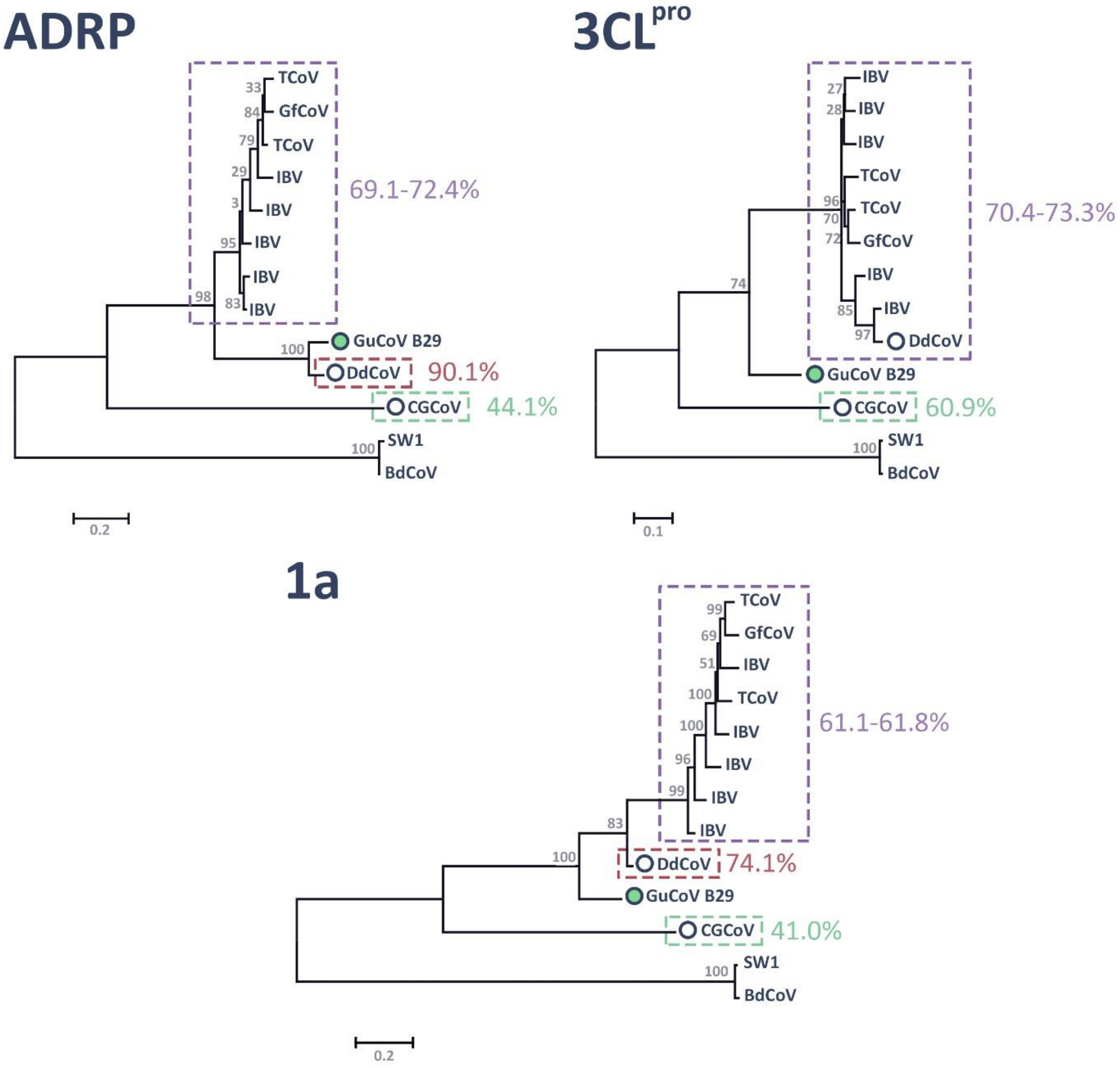
ADRP
TCoV
GfCoV
TCoV
IBV
IBV
IBV
IBV
IBV
69.1-72.4%
GuCoV B29
DdCoV
90.1%
CGCoV
44.1%
SW1
BdCoV
0.2
3CLpro
IBV
IBV
IBV
TCoV
TCoV
GfCoV
IBV
IBV
DdCoV
70.4-73.3%
GuCoV B29
CGCoV
60.9%
SW1
BdCoV
0.1
1a
TCoV
GfCoV
IBV
TCoV
IBV
IBV
IBV
IBV
61.1-61.8%
DdCoV
74.1%
GuCoV B29
CGCoV
41.0%
SW1
BdCoV
0.2

Supplementary Figure S4. Phylogenetic analyses of avian caliciviruses. The trees are based on amino acid sequences of the whole portion of the ORF1 coding for nonstructural proteins (NS) and the capsid protein VP2 of all avian caliciviruses and two mammalian viruses as an outgroup (PeDu: pink-eared duck; Du: duck; Go: goose; Av: avocet; Cr: crane; Tu: turkey; Ch: chicken; Gt: grey teal; Rt: ruddy turnstone; Sd: shelduck; Hu: human; Po: porcine). Recently identified viruses that lack official taxonomic designation are indicated by circles with the viruses identified in this study indicated by filled green circles. In each tree the amino acid identities of DuCV-2 compared to its three closest relatives included in the dotted box are indicated. The trees were built with the maximum likelihood method (Felsenstein, 1981) using MEGA 7 (Kumar et al., 2016) based on the best-fitting model identified by the model test in MEGA (LG model (Le and Gascuel, 2008)). A discrete Gamma distribution was used to model evolutionary rate differences among sites and the rate variation model allowed for some sites to be evolutionarily invariable (for NS only). Branch lengths are proportional to genetic distances as indicated by the scale bar, and the outcome of the bootstrap analysis (Felsenstein, 1985) is shown next to the nodes.

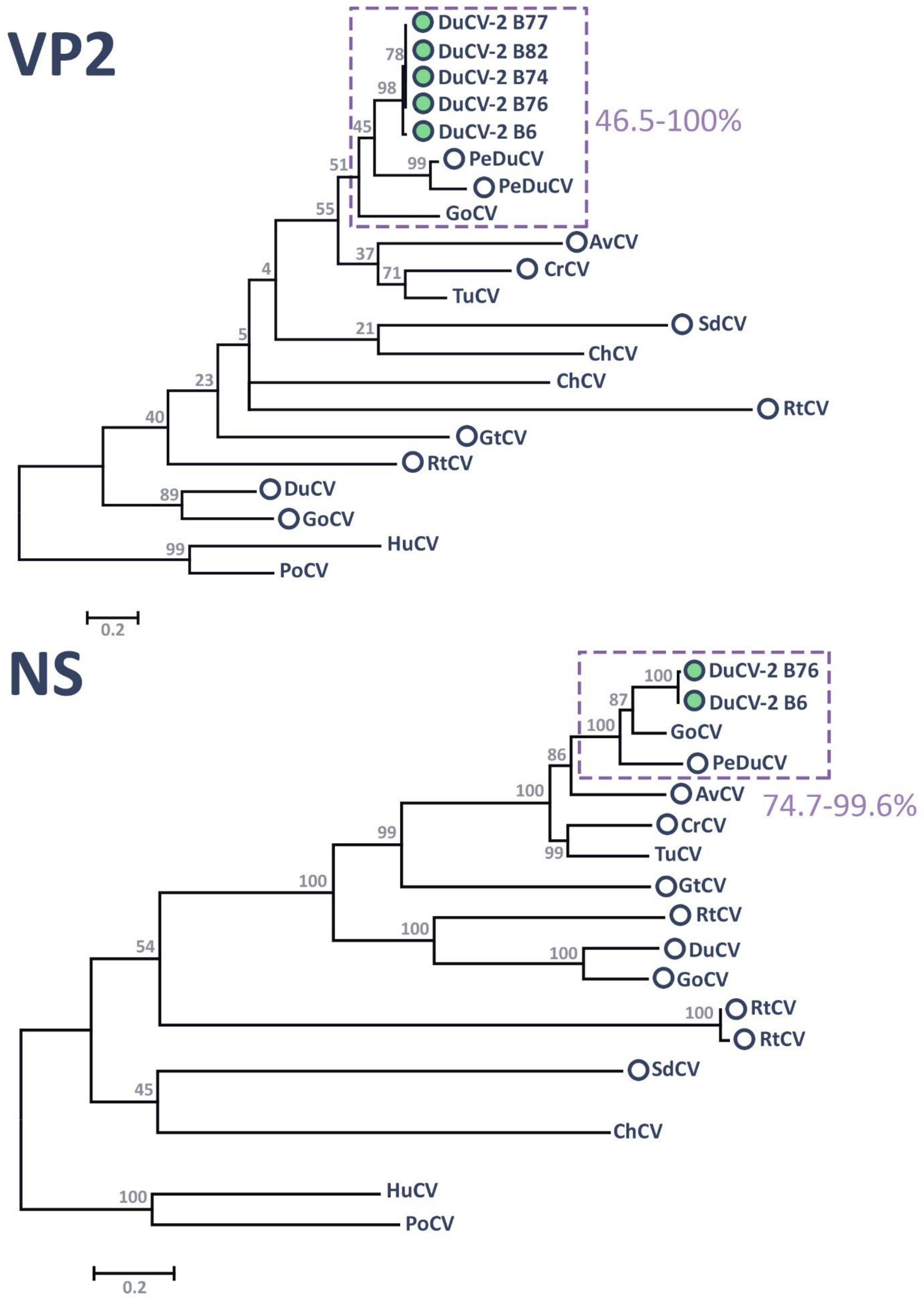
VP2
DuCV-2 B77
DuCV-2 B82
DuCV-2 B74
DuCV-2 B76
DuCV-2 B6
PeDuCV
PeDuCV
GoCV
46.5-100%
AvCV
CrCV
TuCV
SdCV
ChCV
ChCV
RtCV
GtCV
RtCV
DuCV
GoCV
HuCV
PoCV
0.2
NS
DuCV-2 B76
DuCV-2 B6
GoCV
PeDuCV
AvCV
74.7-99.6%
CrCV
TuCV
GtCV
RtCV
DuCV
GoCV
RtCV
RtCV
SdCV
ChCV
HuCV
PoCV
0.2

References

Felsenstein, J. (1981). Evolutionary trees from DNA sequences: a maximum likelihood approach. *J. Mol. Evol.* 17, 368–376.

Felsenstein, J. (1985). Confidence limits on phylogenies: an approach using the bootstrap. *Evolution* 39, 783. doi:10.2307/2408678.

Jones, D. T., Taylor, W. R., and Thornton, J. M. (1992). The rapid generation of mutation data matrices from protein sequences. *Comput. Appl. Biosci.* 8, 275–282. doi:10.1093/bioinformatics/8.3.275.

Kumar, S., Stecher, G., and Tamura, K. (2016). MEGA7: Molecular Evolutionary Genetics Analysis version 7.0 for bigger datasets. *Mol. Biol. Evol.* 33, 1870–1864. doi:10.1093/molbev/msw054.

Le, S. Q., and Gascuel, O. (2008). An improved general amino acid replacement matrix. *Mol. Biol. Evol.* 25, 1307–1320. doi:10.1093/molbev/msn067.

Whelan, S., and Goldman, N. (2001). A general empirical model of protein evolution derived from multiple protein families using a maximum-likelihood approach. *Mol. Biol. Evol.* 18, 691–699. doi:10.1093/oxfordjournals.molbev.a003851.
